# Preventing Preeclampsia With Low-Dose Aspirin: A Systematic Review of Efficacy Across Diverse Populations

**DOI:** 10.7759/cureus.84202

**Published:** 2025-05-15

**Authors:** Rebecca George, Zaheeruddin Sayed, Hamesh Gundala Raja, Asma Qayyum, Izmay Ali, Noman A Jarang, Ishan Solanki, Sabiha Ahmad, Saba Ahmed, Asma Mehdi

**Affiliations:** 1 Obstetrics and Gynecology, Malankara Orthodox Syrian Church Medical College, Kolenchery, IND; 2 Internal Medicine, Chandka Medical College, Larkana, PAK; 3 Internal Medicine, K.A.P. Viswanatham Government Medical College, Tiruchirappalli, IND; 4 Obstetrics and Gynecology, Bukayriyah General Hospital, Al-Bukayriyah, SAU; 5 Internal Medicine, Women Medical and Dental College, Abbottabad, PAK; 6 Internal Medicine, Belarusian State Medical University, Minsk, BLR; 7 Gynecology, Zhejiang University, Hangzhou, CHN; 8 Anesthesia and Critical Care, HSE Kerry Community Services, Kerry, IRL; 9 Surgery, Rawalpindi Medical University, Rawalpindi, PAK

**Keywords:** aspirin resistance, biomarkers, high-risk pregnancies, low-dose aspirin, personalized medicine, preeclampsia prevention, randomized controlled trials

## Abstract

This systematic review evaluates the efficacy of low-dose aspirin (LDA) in preventing preeclampsia in high-risk pregnancies. A comprehensive search of recent randomized controlled trials was conducted, focusing on studies published within the last five years. The review included five studies that investigated LDA at doses ranging from 60 mg to 150 mg, with outcomes measured in diverse populations. Findings indicate that LDA can reduce preeclampsia risk, particularly in specific subgroups such as non-Hispanic white women, but its efficacy is influenced by factors such as aspirin resistance, ethnicity, and biomarker levels. The review also highlights the importance of anti-inflammatory biomarkers like 15-epi-lipoxin A4 and IL-2 in understanding aspirin’s mechanism of action. However, significant variability was observed across studies, suggesting that a personalized approach to aspirin prophylaxis may be necessary. This review underscores the need for future research to address gaps in aspirin efficacy across different populations and to explore biomarker-driven strategies for preeclampsia prevention.

## Introduction and background

Preeclampsia is a hypertensive disorder unique to pregnancy that poses significant risks to maternal and fetal health [1]. Characterized by elevated blood pressure and proteinuria, it is a leading cause of maternal mortality and morbidity worldwide. Preeclampsia affects approximately 2-8% of pregnancies globally, with higher prevalence in low- and middle-income countries [2]. The condition not only compromises maternal health but also increases the risk of preterm birth, low birth weight, and long-term cardiovascular complications in both the mother and child. Despite advancements in obstetric care, preeclampsia remains a clinical challenge due to its unpredictable nature and the limited availability of effective prophylactic strategies [3].

Low-dose aspirin (LDA) has emerged as a promising intervention for preventing preeclampsia in high-risk pregnancies. The preventive effects of aspirin are primarily attributed to its ability to inhibit platelet aggregation and reduce systemic inflammation, both of which play a critical role in the pathophysiology of preeclampsia [4]. In 2014, the U.S. Preventive Services Task Force (USPSTF) recommended the use of LDA for pregnant women at high risk of preeclampsia, which has since been widely adopted in clinical practice [5]. However, the efficacy of LDA varies based on factors such as dosage, timing of initiation, patient demographics, and underlying risk factors. This variability necessitates a comprehensive evaluation of the existing clinical trials to provide evidence-based recommendations for its use [6].

Several randomized controlled trials (RCTs) and cohort studies have investigated the efficacy of LDA in preventing preeclampsia, yielding mixed results. While some studies report significant reductions in preeclampsia incidence and related complications, others show limited benefits. Furthermore, the impact of LDA on maternal and neonatal outcomes across different populations and healthcare settings remains underexplored.

The Population, Intervention, Comparison, Outcome (PICO) framework [7] is an essential tool for structuring a focused research question in systematic reviews. For this review, the population includes pregnant women identified as being at high risk of developing preeclampsia, such as those with a history of preeclampsia, chronic hypertension, multiple gestations, diabetes, or autoimmune disorders. The intervention involves administering LDA, typically 75-150 mg daily, starting in early to mid-pregnancy (before 16 weeks of gestation) as a prophylactic measure against preeclampsia. The comparison is made with placebo or no intervention groups, as well as alternative interventions, to evaluate the relative effectiveness of LDA. The primary outcomes assessed include the incidence of preeclampsia, gestational hypertension, and maternal-fetal complications, while secondary outcomes encompass neonatal outcomes such as preterm birth, low birth weight, and admission to the neonatal intensive care unit. The research question guiding this systematic review is, “What is the comparative efficacy of LDA in preventing preeclampsia and related maternal-fetal complications in high-risk pregnancies compared to placebo or no intervention?” Adhering to the PICO framework allows for a focused and comprehensive evaluation of the existing evidence, identifying both the benefits and limitations of LDA use across diverse clinical scenarios. The insights gained from this review aim to contribute to refining clinical guidelines and improving the quality of care provided to pregnant women at risk of preeclampsia.

## Review

Materials and methods

Search Strategy

The search strategy for this systematic review was designed to comprehensively identify relevant studies assessing the efficacy of LDA in preventing preeclampsia. A structured and systematic approach was employed using major databases, including PubMed, Scopus, and Cochrane Library, to retrieve peer-reviewed articles published within the last five years. Keywords and Medical Subject Headings (MeSH) terms such as “low-dose aspirin”, “preeclampsia prevention”, “randomized controlled trials”, and “high-risk pregnancies” were used to refine the search results. The search was conducted in English-language studies only. The review process followed the Preferred Reporting Items for Systematic reviews and Meta-Analyses (PRISMA) [8] guidelines to ensure transparency and methodological rigor. The PRISMA flowchart was used to document the study selection process, starting with initial identification, screening, eligibility assessment, and final inclusion. Duplicate records were removed, and studies were screened based on predefined inclusion and exclusion criteria. The adherence to PRISMA guidelines strengthens the credibility of this review by providing a transparent framework for identifying, selecting, and appraising relevant literature. A detailed summary of the search strategy used in each database is presented in Table 1.

**Table 1:** Summary of the electronic database search strategies used to identify eligible studies

Database	Search terms used	Filters applied	Records retrieved
PubMed	(“low-dose aspirin” OR “aspirin prophylaxis”) AND (“preeclampsia prevention” OR “preeclampsia”) AND (“randomized controlled trial” OR “RCT”)	English, last five years	198
Scopus	TITLE-ABS-KEY(“low-dose aspirin” AND “preeclampsia” AND “RCT”)	English, last five years	163
Cochrane Library	(“low-dose aspirin” in Title, Abstract, or Keywords) AND (“preeclampsia”) AND (“randomized trial”)	Trials only, English, last five years	90
Total			451

Eligibility Criteria

The eligibility criteria for this systematic review were carefully defined to ensure the inclusion of high-quality studies relevant to assessing the efficacy of LDA in preventing preeclampsia. Only RCTs were included, as they provide the highest level of evidence for evaluating interventions. Studies were eligible if they investigated the use of LDA (dosages between 60 mg and 150 mg) administered to high-risk pregnant women for the prevention of preeclampsia. High-risk populations were defined based on recognized maternal risk factors, including a history of preeclampsia, chronic hypertension, diabetes, multiple gestations, or other relevant comorbidities. Both primary outcomes (incidence of preeclampsia) and secondary outcomes (maternal and neonatal outcomes such as gestational hypertension, preterm birth, and biomarker changes) were considered in the inclusion criteria.

Studies were excluded if they were non-randomized, did not include a placebo or control group, or involved interventions other than LDA. Case reports, reviews, observational studies, and animal studies were also excluded to maintain the review’s focus on high-level evidence from clinical trials. Additionally, articles that did not report detailed outcomes related to preeclampsia prevention or had unclear methodology were excluded. No restrictions were placed on the geographic location of studies, ensuring a diverse representation of populations. However, the review was limited to English-language articles, which may introduce some bias. Furthermore, only studies published within the last five years were included to ensure the relevance of findings to current clinical practice. The strict eligibility criteria ensured that only relevant, high-quality studies were included, providing a robust evidence base for evaluating the effectiveness of LDA in preeclampsia prevention.

Data Extraction

The data extraction process for this systematic review was carried out systematically to ensure accuracy and consistency across all included studies. A standardized data extraction sheet was used to capture key details from each study, including the authors, publication year, study population characteristics, intervention protocols, comparison groups, primary and secondary outcomes, and key findings. The extraction process focused on both clinical outcomes, such as the incidence of preeclampsia and related maternal and neonatal complications, and biomarker analyses, including levels of anti-inflammatory markers like 15-epi-lipoxin A4 and IL-2. Data were independently verified to minimize errors, and any discrepancies were resolved through discussion among the reviewers. Additionally, specific attention was given to identifying the risk of bias within the studies, and this information was recorded during data extraction for subsequent quality assessment. By following a structured extraction process, this review ensured that all relevant data were collected in a comprehensive and reproducible manner, forming a solid foundation for the analysis and synthesis of findings.

Data Analysis and Synthesis

The data analysis and synthesis in this systematic review followed a qualitative approach, focusing on identifying patterns, themes, and consistencies across the included studies to evaluate the efficacy of LDA in preventing preeclampsia. Given the heterogeneity in study designs, populations, and outcomes, a narrative synthesis was employed to integrate findings without performing a meta-analysis. The key outcomes extracted from each study, such as preeclampsia incidence, maternal and neonatal complications, and biomarker levels, were compared to identify recurring trends and discrepancies. The qualitative synthesis highlighted differences in aspirin efficacy based on factors such as ethnicity, dosage, and aspirin resistance. Additionally, the synthesis emphasized the role of biomarkers like 15-epi-lipoxin A4 and IL-2 in understanding aspirin’s mechanism of action. By categorizing and critically analyzing these findings, the review provided a comprehensive narrative that captured the nuances of current evidence, contributing to a more personalized understanding of LDA use in clinical practice.

Results

Study Selection Process

**Figure 1:**
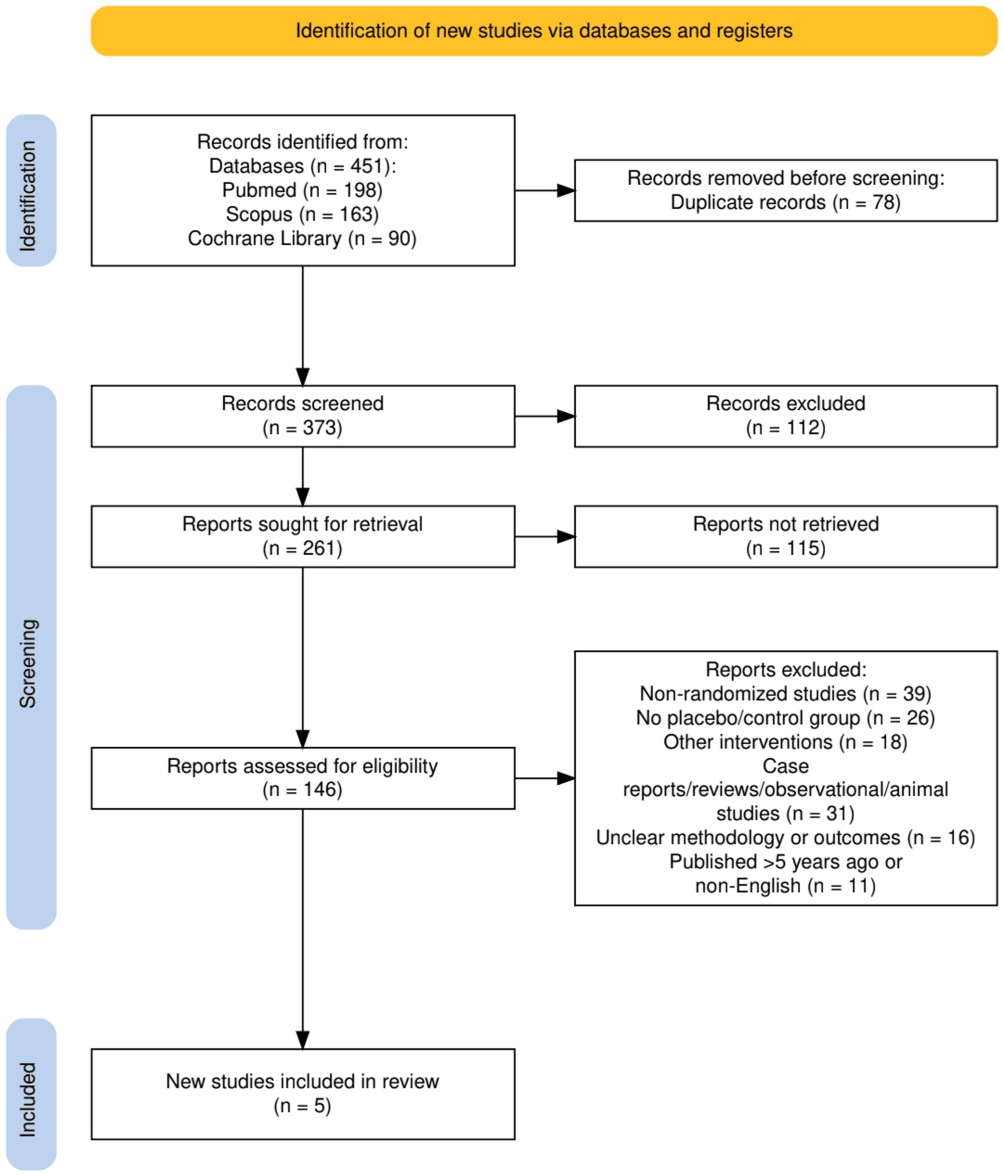
PRISMA flowchart showing the study selection process PRISMA, Preferred Reporting Items for Systematic reviews and Meta-Analyses

The study selection process followed the PRISMA 2020 guidelines and is illustrated in Figure 1. A total of 451 records were identified through database searches: PubMed (n = 198), Scopus (n = 163), and Cochrane Library (n = 90). After removing 78 duplicate records, 373 records remained for screening. Of these, 112 records were excluded based on titles and abstracts. 261 full-text reports were sought for retrieval, but 114 could not be retrieved, leaving 147 reports assessed for eligibility. Following full-text review, 141 articles were excluded for reasons including non-randomized design (n = 39), absence of placebo/control group (n = 26), use of interventions other than LDA (n = 18), being case reports, reviews, observational or animal studies (n = 31), unclear methodology or outcomes (n = 16), or being older than five years or not published in English (n = 11). Ultimately, six studies met the eligibility criteria and were included in the final systematic review.

Characteristics of the Selected Studies

The selected studies included in this systematic review, summarized in Table 2, focused on evaluating the efficacy of LDA in preventing preeclampsia across various high-risk populations. The study populations varied from normotensive nulliparous women to those with chronic hypertension, diabetes, and other risk factors for preeclampsia. The dosage of aspirin ranged from 60 mg to 150 mg daily, with most interventions initiated between 12 and 26 weeks of gestation. Placebo groups were used for comparison in all trials, and the primary outcome assessed was the incidence of preeclampsia. Secondary outcomes included maternal and neonatal complications such as preterm delivery, placental abruption, small for gestational age, and biomarker changes. The studies also explored the role of biomarkers like pregnancy-associated plasma protein A (PAPP-A), placental growth factor (PlGF), and 15-epi-lipoxin A4, along with the impact of aspirin resistance. While some studies demonstrated significant reductions in preeclampsia incidence, particularly in specific subgroups, others reported no significant difference between the aspirin and placebo groups. The heterogeneity in population characteristics and outcomes highlights the complexity of assessing LDA’s efficacy in diverse clinical scenarios.

**Table 2:** Summary of studies evaluating the efficacy of LDA in preventing preeclampsia in high-risk pregnancies LDA, low-dose aspirin; PAPP-A, pregnancy-associated plasma protein A; PlGF, placental growth factor; sTNF-R1, soluble tumor necrosis factor receptor 1; sTNF-R2, soluble tumor necrosis factor receptor 2; TXB2, thromboxane B2

Author and year	Population	Intervention	Comparison	Primary outcomes	Secondary outcomes	Key findings
Tolcher et al. (2020) [9]	Normotensive nulliparous women (low risk) and women with pregestational insulin-treated diabetes mellitus, chronic hypertension, multiple gestations, or a history of preeclampsia (high risk)	60 mg aspirin daily, administered between 13 and 26 weeks of gestation	Placebo	Incidence of preeclampsia	Gestational age at delivery, preterm delivery, placental abruption, small for gestational age, stillbirth, neonatal death	Preeclampsia risk was significantly reduced among non-Hispanic white women in the low-risk trial (P = 0.007). There was no significant reduction among Hispanic, non-Hispanic Black or other groups. In high-risk women, aspirin did not significantly reduce preeclampsia incidence. Increased risk of placental abruption and stillbirth was noted.
Lin et al. (2022) [10]	High-risk pregnant women in China (based on maternal risk factors)	100 mg aspirin daily, administered between 12 and 20 weeks of gestation until 34 weeks	Placebo	Incidence of preeclampsia	Maternal and neonatal outcomes, including postpartum hemorrhage	No significant difference in preeclampsia incidence between the aspirin and control groups (P = 0.924). Postpartum hemorrhage rates were also similar between groups. Subgroup analysis showed no significant differences in preeclampsia incidence based on risk factors.
Rolnik et al. (2024) [11]	Pregnant women at increased risk of preterm preeclampsia, identified using the Fetal Medicine Foundation algorithm	150 mg aspirin daily from before 14 weeks to 36 weeks of gestation	Placebo	Trajectories of serum PAPP-A and PlGF	Biomarker levels at multiple gestational time points	Aspirin had no significant effect on PAPP-A or PlGF trajectories compared to placebo.
Gonzalez-Brown et al. (2021) [12]	Pregnancies at high risk of developing preeclampsia	60 mg aspirin daily, administered between 13 and 26 weeks of gestation	Placebo	Levels of 15-epi-lipoxin A4 (anti-inflammatory biomarker)	Changes in biomarker levels at different gestational periods	Daily LDA significantly increased 15-epi-lipoxin A4 levels compared to placebo. Pregnancies that developed preeclampsia had lower levels of 15-epi-lipoxin A4 compared to those without preeclampsia in the LDA group.
Hernandez et al. (2024) [13]	High-risk pregnant individuals receiving LDA	60 mg aspirin daily	Placebo	Incidence of preeclampsia	Maternal serum biomarker levels (PLGF, IL-2, IL-6, TXB2, sTNF-R1, and sTNF-R2)	LDA resistance was observed in 22.1% of individuals. Mean IL-2 concentrations were significantly lower in LDA-resistant individuals compared to LDA-sensitive individuals. There was no significant difference in preeclampsia prevalence between the LDA and placebo groups.

Quality Assessment

The quality assessment of the included studies was conducted using the Cochrane Risk of Bias 2 (RoB 2) tool [14], focusing on key domains such as the randomization process, deviations from intended interventions, missing data, outcome measurement, and the selection of reported results. As summarized in Table 3, the overall risk of bias was rated as low for most studies, particularly those with well-defined randomization processes and clear outcome reporting. The study investigating the efficacy of LDA based on ethnicity and race was rated low in risk of bias due to its robust methodology and comprehensive reporting. Similarly, the trial conducted in China was deemed low risk, although minor concerns were noted regarding missing data. However, some studies, such as the analysis of aspirin resistance and biomarker trajectories, were rated moderate in risk of bias due to concerns about reporting consistency and sample size limitations. These variations in quality highlight the importance of considering study design and methodology when interpreting the findings, as potential biases could impact the generalizability of the results.

**Table 3:** Quality assessment of included studies evaluating the efficacy of LDA in preventing preeclampsia LDA, low-dose aspirin; RoB 2, Risk of Bias 2

Study	Quality assessment tool	Risk of bias domains assessed	Overall risk of bias	Comments
Low-dose aspirin for preeclampsia prevention: efficacy by ethnicity and race [9]	Cochrane RoB 2	Randomization process, deviations from intended interventions, missing data, outcome measurement, selection of reported results	Low	Well-conducted with clear randomization and outcome reporting
A randomized controlled trial of low-dose aspirin for the prevention of preeclampsia in women at high risk in China [10]	Cochrane RoB 2	Randomization process, deviations from intended interventions, missing data, outcome measurement, selection of reported results	Low	Large sample size and good randomization, but minor concerns about missing data
Aspirin for evidence-based preeclampsia prevention trial: effects of aspirin on maternal serum pregnancy-associated plasma protein A and placental growth factor trajectories in pregnancy [11]	Cochrane RoB 2	Randomization process, deviations from intended interventions, missing data, outcome measurement, selection of reported results	Low to moderate	Comprehensive biomarker analysis, but some concerns about reporting consistency
Low-dose aspirin increases 15-epi-lipoxin A4 in pregnancies at high risk for developing preeclampsia [12]	Cochrane RoB 2	Randomization process, deviations from intended interventions, missing data, outcome measurement, selection of reported results	Moderate	Good biomarker analysis, but small sample size limits generalizability
Aspirin resistance in pregnancy is associated with reduced IL-2 concentrations in maternal serum: Implications for aspirin prophylaxis for preeclampsia [13]	Cochrane RoB 2	Randomization process, deviations from intended interventions, missing data, outcome measurement, selection of reported results	Moderate	Potential concerns regarding aspirin resistance criteria and sample size

Discussion

The findings of this systematic review reveal a mixed efficacy of LDA in preventing preeclampsia across various populations and study designs. In the study by Tolcher et al. [9], a significant reduction in preeclampsia incidence was observed among non-Hispanic white women receiving 60 mg of aspirin daily (P = 0.007), but this effect was not consistent across other ethnic groups, including Hispanic and non-Hispanic Black women. In contrast, Lin et al. [10] conducted a large randomized trial in China with high-risk pregnant women, administering 100 mg of aspirin daily between 12 and 20 weeks until 34 weeks of gestation. The study found no significant difference in preeclampsia incidence between the aspirin group and the placebo group (P = 0.924), indicating that LDA may not universally reduce the risk of preeclampsia in all populations.

Biomarker studies provide additional insights into the potential mechanisms of aspirin in preeclampsia prevention. Rolnik et al. [11] investigated the impact of 150 mg of aspirin on serum PAPP-A and PlGF trajectories in high-risk pregnancies but found no significant differences compared to placebo. Gonzalez-Brown et al. [12], however, reported that daily LDA significantly increased the levels of 15-epi-lipoxin A4, an anti-inflammatory biomarker, suggesting that aspirin’s anti-inflammatory effects may play a role in reducing preeclampsia risk. Despite these promising findings, Hernandez et al. [13] highlighted the issue of aspirin resistance, with 22.1% of the study population being classified as LDA-resistant based on thromboxane B2 levels. These individuals exhibited significantly lower concentrations of IL-2, a biomarker linked to immune modulation, and showed no significant reduction in preeclampsia prevalence compared to placebo (P > 0.05) [15]. Collectively, these findings suggest that while LDA shows potential in reducing preeclampsia risk through anti-inflammatory pathways, its efficacy is influenced by population characteristics, biomarker responses, and aspirin resistance, warranting a more personalized approach to prophylactic aspirin use in pregnancy [16,17].

The findings of this systematic review are generally consistent with existing guidelines that recommend LDA for preeclampsia prevention in high-risk pregnancies, particularly those issued by the USPSTF and the International Federation of Gynecology and Obstetrics. Both guidelines advocate initiating LDA before 16 weeks of gestation to maximize its efficacy [18]. However, this review highlights inconsistencies in aspirin’s effectiveness across different populations, which is less emphasized in existing guidelines. For example, Tolcher et al. [9] reported a significant reduction in preeclampsia among non-Hispanic white women but found no similar effect in other ethnic groups, suggesting that ethnicity may influence aspirin’s efficacy. Current guidelines do not address the potential impact of genetic or racial differences in aspirin metabolism, indicating a gap that future research should address to refine recommendations for specific subgroups [19].

In contrast to some earlier meta-analyses that have shown LDA to consistently reduce the risk of preeclampsia, this review identified studies such as those by Lin et al. [10] and Hernandez et al. [13], which found no significant reduction in preeclampsia incidence in their populations. One possible explanation for this discrepancy is the variability in aspirin resistance among individuals, as highlighted in the Hernandez study. This concept of aspirin resistance, which is influenced by biomarkers like thromboxane B2 and IL-2, is rarely mentioned in existing guidelines but may explain why some high-risk women do not benefit from aspirin prophylaxis [20]. Furthermore, biomarker-focused studies like Gonzalez-Brown et al. [12] add to the growing evidence that LDA's effectiveness may depend on its anti-inflammatory effects rather than solely on its impact on blood pressure. These findings suggest that personalized approaches, incorporating biomarker screening and tailored dosing, could enhance aspirin’s preventive potential, which current guidelines may need to consider in future updates.

The findings of this systematic review highlight important clinical implications for the use of LDA in preventing preeclampsia, suggesting that a more personalized approach may enhance its effectiveness [21]. Current guidelines recommend LDA for high-risk pregnancies without considering factors such as aspirin resistance, genetic variability, or differences in biomarker responses. The evidence from studies, such as those by Gonzalez-Brown et al. [12] and Hernandez et al. [13], emphasizes the potential role of biomarkers like thromboxane B2 and IL-2 in identifying patients who may not respond to aspirin prophylaxis. Clinicians should consider incorporating biomarker screening into routine care for high-risk pregnancies to better predict aspirin response and adjust interventions accordingly. Additionally, the findings suggest that a one-size-fits-all approach to aspirin dosing may be insufficient, particularly in populations with varying genetic backgrounds [22]. Policymakers should update guidelines to account for these individual variations and encourage further research into personalized aspirin prophylaxis to improve maternal and neonatal outcomes.

This systematic review has several strengths, including a comprehensive search strategy that identified recent high-quality RCTs and the use of rigorous quality assessment tools, such as the Cochrane RoB 2 tool, to evaluate the included studies. The review covers diverse populations and investigates both clinical outcomes and biomarker trajectories, providing a broad perspective on the efficacy of LDA in preventing preeclampsia. However, there are some limitations that should be acknowledged. The included studies vary in aspirin dosages, timing of intervention, and population characteristics, which may contribute to inconsistencies in the findings. Additionally, language restrictions and the exclusion of non-English studies may limit the generalizability of the results. The review also faced limitations related to small sample sizes in some trials and potential publication bias, where studies with negative outcomes may be underreported.

Bias and heterogeneity are important factors to consider when interpreting the findings of this review. Variability in study designs, including differences in baseline risk factors, dosing regimens, and outcome definitions, introduces heterogeneity that may affect the comparability of results across studies. For example, differences in the definition of preeclampsia and variations in how biomarkers were measured could impact the observed outcomes. Potential biases, such as selection bias and reporting bias, may have influenced the results, particularly in studies where compliance with aspirin therapy was not consistently monitored. Furthermore, the issue of aspirin resistance, which was identified in some studies, highlights an area of variability that may account for the differing efficacy of LDA across populations [23]. Addressing these biases and heterogeneities in future research is essential to better understand the role of aspirin in preventing preeclampsia and to develop more targeted clinical interventions.

Future research should focus on addressing key gaps identified in this systematic review, particularly the variability in aspirin efficacy across different populations and the impact of aspirin resistance on preeclampsia prevention. Studies should explore the underlying mechanisms of aspirin resistance, including genetic factors and biomarker profiles, to better understand why some individuals do not respond to LDA therapy. Research into personalized aspirin prophylaxis, incorporating biomarker screening such as thromboxane B2 and IL-2 levels, is essential to optimize outcomes for high-risk pregnancies. Additionally, future trials should evaluate the effectiveness of varying aspirin dosages and initiation times to identify the most beneficial regimen for different subgroups. Long-term studies are also needed to assess the impact of LDA on maternal and neonatal outcomes beyond the immediate postpartum period [24]. Finally, addressing the lack of data from diverse populations, particularly in low- and middle-income countries, would enhance the generalizability of findings and support more inclusive, evidence-based guidelines for preeclampsia prevention.

## Conclusions

This systematic review highlights that LDA has potential benefits in reducing the risk of preeclampsia in high-risk pregnancies, but its efficacy is influenced by several factors, including ethnicity, aspirin resistance, and biomarker responses. While some studies demonstrated significant reductions in preeclampsia incidence, particularly in specific subgroups such as non-Hispanic white women, others found no overall benefit in broader populations. The review also underscores the importance of anti-inflammatory pathways, as indicated by increased 15-epi-lipoxin A4 levels in response to aspirin, and the need to address aspirin resistance through personalized approaches. However, the variability in outcomes suggests that a one-size-fits-all aspirin regimen may not be universally effective. Future guidelines should consider incorporating biomarker-based screening and tailored dosing strategies to improve LDA’s efficacy. Overall, this review reinforces the role of aspirin in preeclampsia prevention while highlighting the need for more personalized, evidence-based approaches in clinical practice.
